# Characterization and Identification of a Novel Torovirus Associated With Recombinant Bovine Torovirus From Tibetan Antelope in Qinghai-Tibet Plateau of China

**DOI:** 10.3389/fmicb.2021.737753

**Published:** 2021-09-06

**Authors:** Xiaoyi Dai, Shan Lu, Guobao Shang, Wentao Zhu, Jing Yang, Liyun Liu, Jianguo Xu

**Affiliations:** ^1^Department of Pathogenic Biology, School of Basic Medical Sciences, Southwest Medical University, Luzhou, China; ^2^State Key Laboratory of Infectious Disease Prevention and Control, National Institute for Communicable Disease Control and Prevention, Chinese Center for Disease Control and Prevention, Beijing, China; ^3^Shanghai Public Health Clinical Center, Fudan University, Shanghai, China; ^4^Research Units of Discovery of Unknown Bacteria and Function, Chinese Academy of Medical Sciences, Beijing, China; ^5^Haixi Prefecture Center for Disease Control and Prevention, Qinghai, China

**Keywords:** novel torovirus, Tibetan antelope, RNA sequencing, natural reservoir host, cross-species transmission

## Abstract

Toroviruses (ToVs) are enteric pathogens and comprise three species, equine torovirus (EToV), bovine torovirus (BToV), and porcine torovirus (PToV). In this study, a novel torovirus (antelope torovirus, AToV) was discovered from fecal samples of Tibetan antelopes (*Pantholops hodgsonii*) with viral loads of 2.10×10^9^ to 1.76×10^10^ copies/g. The genome of AToV is 28,438 nucleotides (nt) in length encoding six open reading frames (ORFs) with 11 conserved domains in pp1ab and a putative slippery sequence (_14171_UUUAAAC_14177_) in the overlapping region of ORF1a and ORF1b. Phylogenetic analysis illustrated strains of AToV form a unique clade within ToVs and comparative analysis showed AToV share relatively low sequence identity with other ToVs in six ORFs (68.2–91.6% nucleotide identity). These data suggested that AToV represents a novel and distinct species of ToVs. Based on the M genes, evolutionary analysis with BEAST of AToV and other ToVs led to a most recent common ancestor estimate of 366years ago. Remarkably, recombination analysis revealed AToV was the unknown parental ToV that once involving in the recombinant events of HE genes of two Dutch strains of BToV (B150 and B155), which indicated that AToV occurred cross-species transmission and existed both in the Netherlands and China. This study revealed a novel torovirus, a natural reservoir host (Tibetan antelope) of toroviruses for the first time, and appealed to further related studies to better understand the diversity of toroviruses.

## Introduction

Toroviruses (ToVs) primarily infect and cause enteric disease and diarrhea in ungulates, especially equine, bovine, and porcine. Toroviruses once belonged to genus *Torovirus*, subfamily Torovirinae, family Coronaviridae, order Nidovirales, then, were reclassified into subgenus Renitovirus, genus *Torovirus*, subfamily Torovirinae, family Tobaniviridae, suborder Tornidovirineae, order Nidovirales according to the Virus Taxonomy: 2018b Release (MSL #34; Hu et al., 2019).^1^ Toroviruses exhibit polymorphisms of spherical, kidney-shaped, and rod-shaped enveloped virions with 100–140 nm in diameter. The genomes of toroviruses are large (~28kb), single-stranded, and positive-sense RNAs. The genomic organization of torovirus is similar to that of coronavirus with non-translated regions (NTRs) locate at both the 5' and 3' termini, two large and overlapping open reading frames (ORFs), ORF1a and ORF1b, which together encode replicase polyprotein (pp1ab), and four small open reading frames corresponding to the S, M, HE, and N genes, which encode the structural proteins: spike protein, membrane protein, hemagglutinin-esterase, and nucleocapsid protein (Draker et al., 2006; Dhama et al., 2014).

*Renitovirus* is the only subgenus in the monotypic genus *Torovirus* including three virus species: equine torovirus (EToV), bovine torovirus (BToV), and porcine torovirus (PToV). human torovirus (HToV) was excluded from the genus *Torovirus* (Hu et al., 2019). EToV, also called as BEV (Berne virus), has been considered as the prototype species of the genus *Torovirus*. In 1972, EToV was initially isolated in cell culture from a rectal swab obtained from a horse with diarrheic and hepatic diseases in Berne, Switzerland (Weiss et al., 1983). BToV, also known as BRV (Breda virus), was later observed by electron microscopy in 1979 from feces of 5-day-old calf with acute enteritis in a dairy farm in Breda, Iowa, United States (Woode et al., 1982). PToV was initially detected in 1998 from the feces of piglets in the Netherlands using immunoelectron microscopy. Sequence alignments revealed that the N gene of the PToV only shares 68.7 and 68.3% nucleotide identity with N genes of previously discovered BToV and EToV, respectively (Kroneman et al., 1998). Both BToV and PToV have been reported worldwide including North and South America, Europe, Asia, and South Africa (Dhama et al., 2014; Hu et al., 2019).

Intertype recombination events often occurred between BToV and PToV at regions of the 5'-NTR, ORF1a, ORF1b, HE gene, N gene, and 3'-NTR. Even some BToV and PToV strains carried chimeric HE genes, which were demonstrated deriving from recombination events among BToV, PToV, and unknown ToVs (Smits et al., 2003; Cong et al., 2013; Ito et al., 2016). Moreover, inter-order recombination events were found between ToV (order Nidovirales) and enterovirus G (order Picornavirales). The genome of porcine enterovirus G detected in feces of diarrheal pigs in farms of China, South Korea, Japan, United States, and Belgium was demonstrated inserting with a gene encoding a papain-like cysteine protease of ToV at the junction site of 2C and 3A genes (Conceição-Neto et al., 2017; Knutson et al., 2017; Shang et al., 2017; Tsuchiaka et al., 2018; Wang et al., 2018; Lee and Lee, 2019).

With the development of high-throughput sequencing, a few new toro-like viruses within the family Tobaniviridae were discovered with whole or partial genome sequences in recent years, such as Guangdong chinese water snake torovirus and Guangdong red-banded snake torovirus (Shi et al., 2018). A *Tobaniviridae* phylogenetic analysis showed that Guangdong chinese water snake torovirus does not cluster with toroviruses within the subfamily Torovirinae and clusters with members of the subfamily Serpentovirinae (Ferron and Debat, 2020). Recently, full genomes of two unclassified toroviruses, goat torovirus (GToV, accession number NC_034976) and Torovirus sp. (ToV sp., accession number MK521914) derived from *Capra hircus* and *Sarcophilus harrisii*, respectively, were recorded in Genbank, but there is no further related research about these toroviruses. In this study, we discovered a novel species of torovirus (antelope torovirus, AToV) from Tibetan antelopes in Qinghai-Tibet plateau of China by RNA sequencing (RNA-seq). Remarkably, our study showed the newly found AToV had been involved in the recombination events of the chimeric HE genes of BToV B150 and B155, which had been identified in Dutch veal calf farms (Smits et al., 2003).

## Materials and Methods

### Sample Collection

Fecal samples of Tibetan antelopes were collected in 2014 from Hoh Xil National Nature Reserve, which is located at the Qinghai-Tibet plateau and administratively belongs to Qinghai Province in China. The excreted feces were picked up directly after Tibetan antelopes moved away. A total of 505 fecal samples were collected from five sites, including 73 samples were collected from site A at an altitude of 4479.5m (36°41' N, 94°92' E), 57 samples were collected from site B at an altitude of 4514.4m (35°37' N, 93°44' E), 162 samples were collected from site C at an altitude of 4561.7m (35°51' N, 93°73' E), 147 samples were collected from site D at an altitude of 4614.6m (35°55' N, 93°86' E), and 66 samples were collected from site E at an altitude of 4693.5m (35°59' N, 94°03' E). The feces were collected in 50-ml sterile tubes with viral transport medium, stored at −20°C in a freezer, and transported to the laboratory in Beijing, and then transferred and stored in a −80°C freezer until use. The fecal samples were collected in a non-invasive way, which was approved by Wildlife Protection Agents of Qinghai.

### RNA Extraction and RNA Sequencing

Total RNA was extracted from each fecal sample using QIAamp RNA Minikit (Qiagen, Hilden, Germany) and eluted in 50μl RNase-Free Water. The extractions obtained from 160 fecal samples were randomly chosen and merged into three pools for RNA sequencing. Total RNA was initially processed as follows: after DNase I digestion, rRNA was removed by Ribo-Zero Magnetic Gold Kit (Human/Rat/Mouse) and Ribo-Zero Magnetic Gold Kit (Bacterial; Epicentre, Madison, United States). The remaining RNA was used to construct RNA-seq library according to the protocol provided by Illumina (Illumina, Sandiego, United States). Briefly, RNA was purified and enriched using oligo (dT) magnetic beads and then fragmented and reverse transcribed into cDNA. After the synthesis of the cDNA, ends repair was performed, followed by adenylation of the 3' end, ligation of sequencing adapter, and quantification using Agilent 2,100 Bioanalyzer and ABI StepOnePlus Real-time PCR system. Pair-end (125bp) sequencing was performed on Hiseq 2,500 platform (Illumina, Sandiego, United States). The library construction and sequencing procedures were performed in BGI Tech (Shenzhen, China). The resulting sequencing reads were analyzed as previously described (Dai et al., 2018).

### RT-PCR Screening

AToV screening for 505 fecal samples was performed by semi-nested RT-PCR. Specific primers were designed based on contigs obtained by RNA sequencing to amplify a 390-bp fragment of the N gene. PrimeScript One Step RT-PCR kit (TaKaRa, Japan) was used for the first reaction. Two microliter of total RNA of each fecal sample was added to an RT-PCR mixture (50μl) included 2μl PrimeScript one Step Enzyme Mix and 1μl (20μM) of each outer primer (F1: 5'-TATGCCTTTTCAACCACCAAC-3', R1: 5'- TGAGACGTTTCATCAGTGGC-3'). After Reverse transcription at 50°C for 30min and initial denaturation at 94°C for 2min, 30cycles of amplification (94°C for 30s, 51°C for 30s, and 72°C for 30s) were performed, followed by 72°C for 10min for the final extension. Subsequently, 5μl of the first-round products were used as the template of the second reaction. In this round, a PCR mixture (20μl) included 2.5U ExTaq DNA polymerase (TaKaRa, Japan) and 1μl (20μM) of each inner primer (F2: 5'- ATGCCTGTTCAGTATCCTTTG-3', R1). After 94°C for 5min, 30cycles of amplification (94°C for 30s, 50°C for 30s, and 72°C for 30s) were performed, followed by 72°C for 10min for the final extension. Products of the second reaction were gel-purified using a QIAquick gel extraction kit (Qiagen, Hilden, Germany) and sequenced using the ABI prism 3700 DNA Analyzer (Applied Biosystems, Foster City, CA, United States) by Sanger method. The sequences of gel-purified products were compared with the sequences of the N gene of AToV.

### Complete Genome Sequencing

To determine the full genome of AToV, a set of specific primers were designed (Supplementary Table S1) based on contigs obtained by RNA sequencing. The total RNA of the AToV-positive fecal sample was used as the template. All RT-PCR amplifications were performed using PrimeScript One Step RT-PCR kit and ExTaq DNA polymerase (TaKaRa, Japan). The terminal ends of the genome were confirmed using a 5'/3'RACE Kit (Roche, Switzerland). The amplicons were sequenced after ligation into the pGEM-T vector (Promega, United States). Sequences were assembled with SeqMan (version 7.1.0) to produce the full-length virus genome sequences.

### Genome Analysis

Open reading frames (ORFs) were predicted by genome comparison of AToV and three annotated ToVs (accession numbers: AY427798, KM403390, and JQ860350). The predicted amino acid sequences of conserved replicase domains of pp1ab of AToV were identified by aligning with annotated pp1ab of other ToVs (accession numbers: P0C6V7 and P0C6V8) and reconfirmed using PSI BLAST. The secondary structure of RNA pseudoknot was predicted using Hotknots online software (Ren et al., 2005). Nucleotide and amino acid sequences were aligned using the ClustalW within the BioEdit software (version 7.1). Phylogenetic analysis was conducted by the maximum likelihood method using MEGA7.0 with 1,000 bootstrap replications. Bootscanning analysis was performed to detect potential recombination events by using an alignment of nucleotide sequences of the M-HE-N (gaps are excluded) segments of BToV B150 (the query), BToV Breda1, PToV Markelo, and AToV Qinghai1 with the SimPlot software. The following parameters were used in bootscanning analysis: window size, 200bp; step size, 20bp; NJ method under Kimura’s two-parameter correction with 100 bootstrap replicates; transition\transversion ratio, 2.0; the threshold for the bootscan was set at 60%.

### Real-Time Quantitative RT-PCR

Real-time RT-PCR was performed on each AToV-positive fecal sample. A forward primer (5'-TGTTTTGTGACCAATCTCTTTACCC-3') and a reverse primer (5'- ACAATAAAAATCAGGTTGGCGGCTA-3') were designed based on sequences of 5'UTR region of AToV genome. Using procedures as follows: the extracted total RNA was diluted 10 times to minimize the interference of background DNA, then, the diluted RNA was reverse transcribed with 1μl (10μM) reverse primer using a PrimeScript RT reagent Kit with gDNA Eraser (Perfect Real Time; TaKaRa, Japan) to produce cDNA. cDNA was amplified with SYBR Premix Ex Taq II kit (Tli RNaseH Plus; TaKaRa, Japan). Concisely, 25μl of a reaction mixture included 2μl cDNA, 10μM of each forward and reverse primers were thermal cycled at 95°C for 30s, followed by 40cycles of 95°C for 5s, 63°C for 30s and 72°C for 30s, using a Rotor-GeneQ cycle (Qiagen, Hilden, Germany). At the end of the assay, PCR products were subjected to melting curve analysis (65 to 95°C, 0.5°C/s) to confirm the specificity of the assay. For quantitation, a reference standard curve was constructed using the pGEM-T Vector (Promega, United States) containing the target sequence.

### Evolutionary Analysis

The mean time of the most recent common ancestor (tMRCA) was estimated based on complete M genes sequence data using the Bayesian Markov Chain Monte Carlo (MCMC) approach employed by BEAST package (version 1.8.0; Drummond and Rambaut, 2007). The jModeltest (version 2.1.10) software was used to estimate the best-fit nucleotide substitution model according to the Akaike information criterion (AIC), with GTR+I+G as the best substitution model (Posada, 2008). Constant size under a relaxed clock model (uncorrelated exponential) was adopted based on Bayes factor analysis (Supplementary Tables S2 and S3). The MCMC analysis was performed with 60 million generations and sampled every 10,000 generations with 10% burn-in. Convergence of parameters was assessed on the basis of the ESS reaching values >200 using Tracer software (version 1.5). Maximum clade credibility (MCC) trees were subsequently generated after 10% burn-in using Tree Annotator and viewed by FigTree. Virus strains used in this study are listed in Supplementary Table S4.

### Electron Microscopy

Positive fecal sample was diluted to 20% suspensions in phosphate-buffered saline (PBS) and centrifuged at 9,000rpm for 10min. The supernatant was collected and further centrifuged at 12,400rpm for 10min. The supernatant was collected again and centrifuged at 100,000 × *g* for 60min. The sediment was collected, resuspended, and subjected to negative staining. The viral particles were observed using transmission electron microscopy (TECNAI 12, FEI, Blackwood, NJ) with an acceleration voltage of 80kV.

### Nucleotide Sequence Accession Numbers

The complete genome sequences of AToV Qinghai1, Qinghai2, and Qinghai3 were submitted to the GenBank with accession numbers MZ438674, MZ438675, and MZ438676, respectively.

## Result

### Identification of AToV Genome From Tibetan Antelope

The RNA extractions of 160 fecal samples of Tibetan antelopes were merged into 3 pools to construct 3 libraries for RNA sequencing. 332,781 and 306,169 *Tobaniviridae* family-related reads were yielded from library 1 (a total of 134,806,135 reads) and library 3 (a total of 248,668,776 reads), respectively. Six contigs were assembled from these reads with a length range of 125–28,438 nt and showed 78–91% amino acid identity to BToV Breda1. Three of these contigs are longer than 10,000nt, one (28,438nt) from library 1 and two (10,504nt and 17,590nt) from library 3.

**Figure 1:**
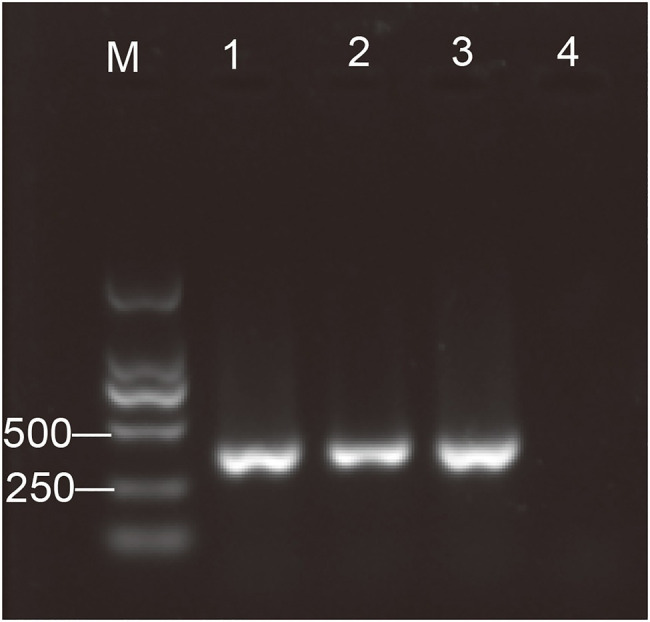
Detection of the antelope torovirus (AToV) by semi-nested RT-PCR. One percent of agarose gel electrophoresis. Lanes: M, Marker, DL2000; 1–3, AToV-positive fecal samples; and 4, negative control. Specific amplicon for the antelope torovirus (AToV) corresponds to a size of 390bp.

Thus, the existence of AToV in fecal samples of Tibetan antelopes was revealed by RNA sequencing. Three AToV-positive fecal samples were found from site C (35°51' N, 93°73' E) and site E (35°59' N, 94°03' E) by detection of the semi-nested RT-PCR method (Figure 1). Two derive from 160 samples that were used for RNA sequencing and one is from the left 345 fecal samples. Full-length genome sequences of the three strains, Qinghai1, Qinghai2, and Qinghai3 (corresponding fecal sample ID 394, 61, and 419, respectively), were obtained by a combination method of RNA-seq, RT-PCR, and RACE. RNA-seq data have already covered the full genome of Qinghai1 and 98% genome of Qinghai2. The three strains of AToV shared the same genome length with 99% identity with each other. The amounts of AToV RNAs detected by quantitative real-time RT-PCR are 2.10×10^9^, 9.30×10^9^, and 1.76×10^10^ copies/g in the three positive fecal samples.

### Genomic Characterization

**Figure 2:**
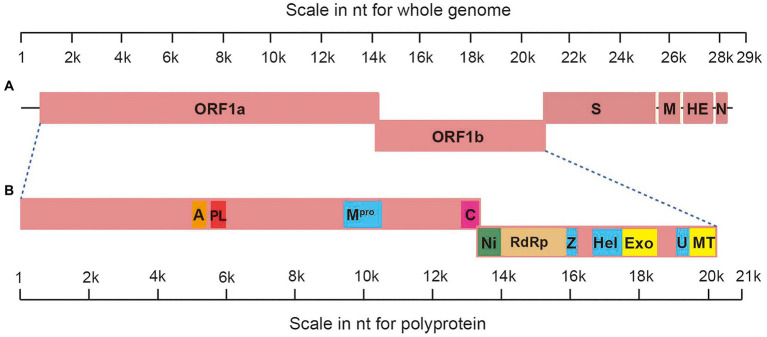
Genomic organization of AToV. **(A)** Schematic representation of AToV genome; **(B)** schematic representation of pp1ab. Conserved domains are highlighted as follows: a represents ADRP, residues V1645-C1773; PL represents PLP, residues G1820-L1968; M^pro^, residues S3131-Q3417; C represents CPD, residues Q4269-D4439; Ni represents NiRAN, residues F4440-R4650; RdRp, residues P4651-Q5233; Z represents ZBD, residues S5284-L5365; Hel, residues V5538-V5813; Exo represents ExoN, residues S5842-V6171; U represents NendoU, residues G6316-Q6465; MT, residues R6466-H6730.

**Figure 3:**
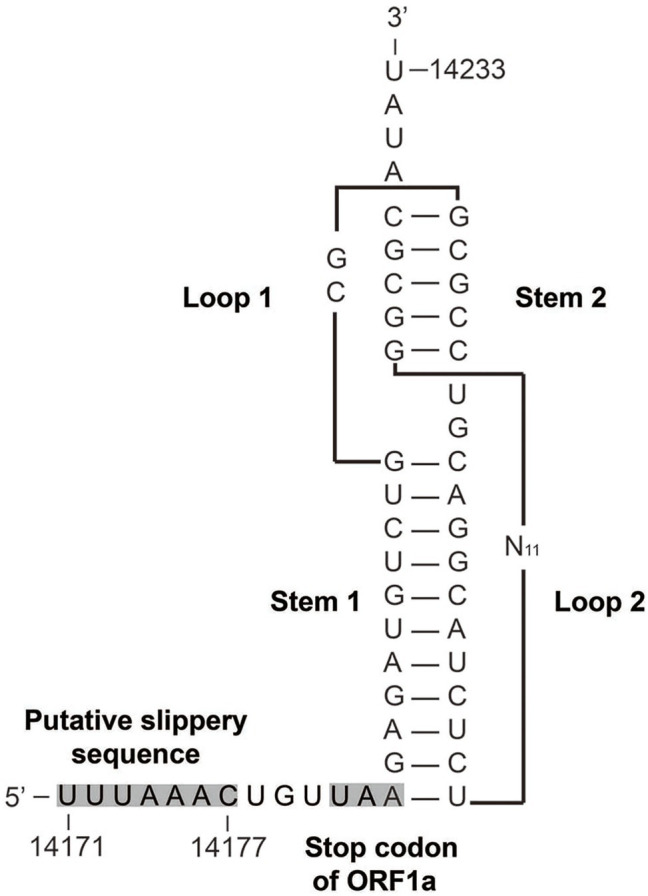
Predicted structure of the AToV ribosomal frameshifting element. The putative slippery sequence and the ORF1a translation termination codon are shaded in gray. N_11_ indicated 11 unshown nucleotides.

The genome of AToV is a 28,438 nucleotides RNA. The G+C content is 37%, which is slightly lower than that of BToV (38%) and higher than that of PToV (35%). The AToV genome consists of six open reading frames (ORFs), an 854-nt NTR at 5' end, and a 195-nt NTR at 3' end common to other ToVs (Figure 2A). ORF1a (13,329nt) and ORF1b (6,876nt) are partially overlapped and locate at the 5'-terminal of the genome, almost cover 70% of the genome. The expression of the huge replicase polyprotein (pp1ab) encoded together by ORF1a and ORF1b is translated upon a ribosomal frameshifting event, which requires two elements, namely a specific seven-nucleotide slippery sequence and an RNA pseudoknot (Brierley, 1995). A putative slippery sequence, _14171_UUUAAAC_14177_, was just found upstream of the AToV ORF1a translation stop codon, and the sequence downstream of the putative slippery sequence could be modeled into an RNA pseudoknot structure with two loops and two stems (Figure 3). These two elements are very similar to those predicted for EToV and BToV (Snijder et al., 1990; Draker et al., 2006). Ten predicted domains (Figure 2B) are identified in pp1ab of AToV including ADP-ribose 1-phosphatase (ADRP), papain-like protease (PLP), 3C-like main protease (M^pro^), cyclic phosphodiesterase (CPD), RNA-dependent RNA polymerase (RdRp), Zn-binding domain (ZBD), Helicase (Hel), 3'-to 5' exoribonuclease domain (ExoN), nidoviral uridylate-specific endoribonuclease (NendoU), and ribose 2'-O-methyltransferase (MT) by aligning with annotated pp1ab of other ToVs and searching result of PSI BLAST. A new domain, conserved in nidoviruses and is N-terminally adjacent to the RdRp, named as nidovirus RdRp-associated nucleotidyl-transferase (NiRAN; Lehmann et al., 2015) has been discovered in AToV as well. The positions of these domains have been described in the figure legend of Figure 2. The main proteinase M^pro^, which mediates the processing steps of the central and C-terminal portions of nidovirus replicase polyprotein, has been identified in BToV and EToV as a serine protease due to the presence of a serine nucleophile in the catalytic triad His-Glu-Ser (Draker et al., 2006). Alignment results revealed that M^pro^ of AToV is also a serine protease with predicted catalytic triad His-Glu-Ser similar to BToV and EToV. Downstream of ORF1b, as ordered from 5' to 3', there are S (4,749nt), M (702nt), HE (1,254nt), and N (489nt) genes (Figure 2A). ORF1b and S gene are partially overlapped, and there are three short non-coding sequences (gaps) between S-M, M-HE, and HE-N with lengths of 28nt, 17nt, and 43nt, respectively.

We conducted a comparative analysis of six ORFs among AToV, BToV, EToV, PToV, and unclassified toroviruses GToV and ToV sp. (Table 1). ORFs of ToV strains used for comparative analysis are non-recombinant excluded HE genes of BToV B150 and B155. For ORF1a, ORF1b, and M gene, AToV Qinghai1 shared 68.8–89.4% nucleotide identity and 76.1–92.3% amino acid identity with that of other ToV strains. Sequence identity of S, HE, and N genes between AToV Qinghai1 and other ToV strains (except BToV B150 and B155) were<80% at both nucleotide and amino acid levels. Surprisingly, the HE gene of AToV Qinghai1 shared high sequence identity (91.7–92.0% nucleotide identity and 94.8% amino acid identity) with recombinant HE genes of BToV B150 and B155. A previous study showed that the HE genes of BToV B150 and B155 resulted from recombination events. About 20% of the HE gene sequences of BToV B150 and B155 were derived from a BToV parent and a PToV parent, and the left 80% were derived from an unknown ToV parent. The HE gene sequences of BToV B150 and B155 were 99.0% identical to each other and shared 66.7–73.6% nucleotide identity with that of other BToV and PToV strains (Smits et al., 2003). The high sequence identity between HE genes of BToV B150, B155, and AToV Qinghai1 revealed that AToV is likely to be the unknown ToV parent.

**Table 1:** Pairwise nucleotide and amino acid sequences comparisons of six open reading frames (ORFs) of antelope torovirus (AToV) Qinghai1 and other Torovirus (ToV) strains.

ORFs	Bovine torovirus (BToV)	Porcine torovirus (PToV)	Equine torovirus (EToV)	Goat torovirus (GToV)	ToV sp.
Nucleotide identity (%)					
1a	89.4	75.6–77.5	68.8	91.6	88.8
1b	80.8	86.3–86.4	80.4	80.9	81.0
S	74.3–74.5	74.7–75.1	72.4	75,2	75.2
M	77.8–78.6	80.6–82.3	78.1	79.2	78.7
HE	71.8–74.2 91.7–92.0	69.8–71.6		72.5	72.4
N	68.2	75.2–77.6	67.0	70.1	69.8
Amino acid identity (%)					
1a	91.3	76.6–77.1	77.5	91.0	90.0
1b	88.0	92.3	86.7	88.1	85.6
S	78.0–78.5	76.4–77.1	73.9	78.8	78.9
M	89.7–91.5	89.7–90.6	88.0	90.1	91.0
HE	71.4–74.6 94.8	66.1–71.7		72.3	70.2
N	61.3	75.6–78.0	61.3	67.9	67.3

*The complete sequences of HE gene of EToV Berne are not available in Genbank. ToV strains used for pairwise comparison are listed in* *Supplementary Table S4* *. The identity between AToV Qinghai1, BToV B150, and B155 are provided.*

### Phylogenetic and Evolutionary Analyses

**Figure 4:**
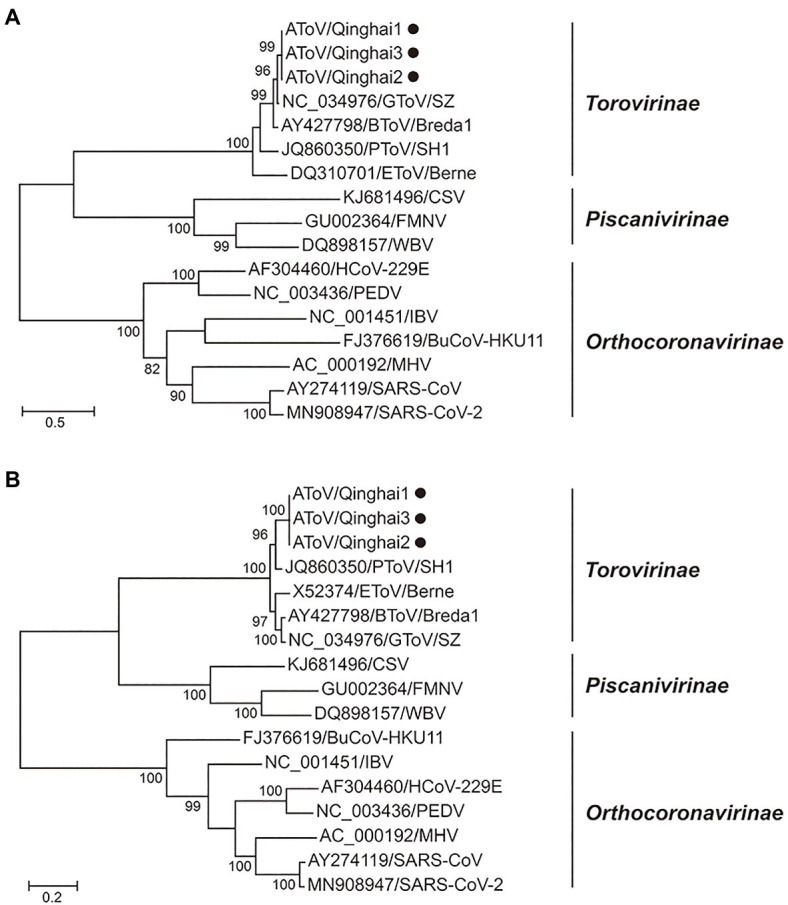
Phylogenetic relationship of toroviruses, piscaniviruses, and coronaviruses within the order Nidovirales. **(A)** The phylogenetic tree of ORF1a; **(B)** The phylogenetic tree of ORF1b. Strains of AToV were marked with black circles. Phylogenetic trees were constructed using MEGA 7.0 by the maximum likelihood method (1,000 bootstrap replications). Bootstrap values (>70%) are shown at the branches. Scale bars below indicate the amino acid substitutions per site.

**Figure 5:**
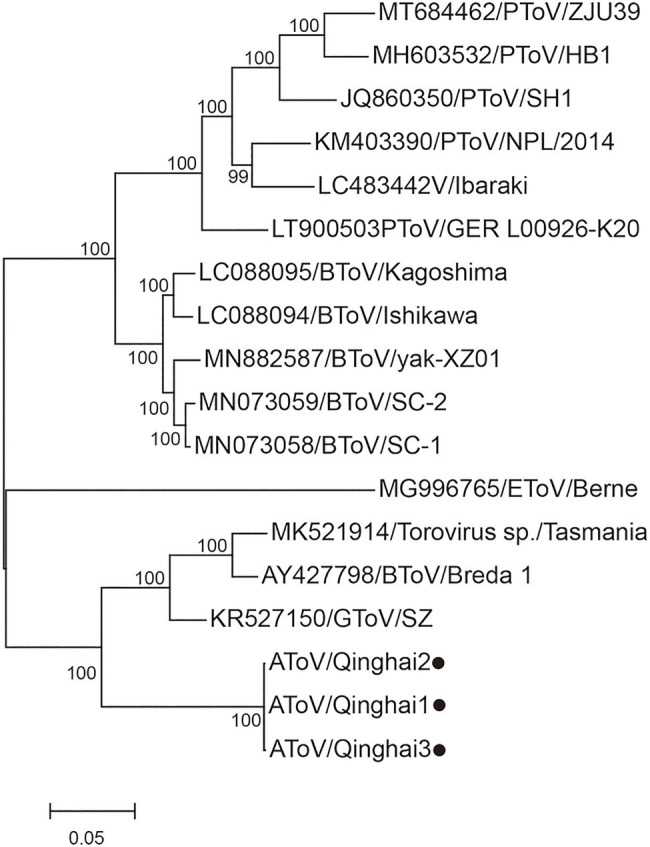
Phylogenetic analysis of the complete genomic nucleotide sequence of all ToVs. Strains of AToV were marked with black circles. The tree was constructed using MEGA 7.0 by the maximum likelihood method (1,000 bootstrap replications). Bootstrap values (>70%) are shown at the branches. The scale bar below indicates the nucleotide substitutions per site.

Phylogenetic trees were constructed using the amino acid sequences of the ORF 1a and ORF 1b of representative virus species of subfamilies Torovirinae, Piscanivirinae, and Orthocoronavirinae. In the two phylogenetic trees, three strains of AToV were clustered with toroviruses within the subfamily Torovirinae (Figure 4). Phylogenetic tree based on nucleotide sequences of complete genomes of three strains of AToV and ToVs, which are available in Genbank showed strains of AToV formed a unique clade within ToVs (Figure 5).

**Figure 6:**
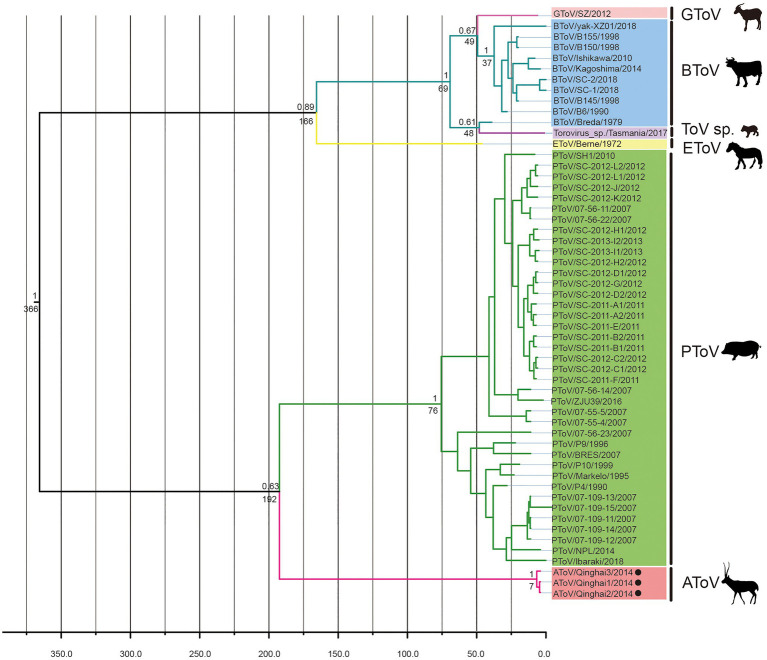
The Bayesian maximum clade credibility tree of M genes of ToVs. The year of sampling, strain name, and virus species are on the tip labels. Node labels indicated the most recent common ancestor (tMRCA) and the posterior probabilities. Strains of AToV were marked with black circles.

BEAST was used to estimate the tMRCA of toroviruses, based on complete nucleotide sequences of the M genes (702nt). Maximum clade credibility (MCC) tree was constructed with 55 strains of ToVs, including 10 strains of BToV, one strain of EToV, one strain of GToV, three strains of AToV, one strain of ToV sp., and 39 strains of PToV (Figure 6). The most recent common ancestor of AToV and other ToVs was estimated at 366 (95% HPD=124–1835) years ago. AToV and PToV were diverged about 192 (95% HPD=66–431) years ago. The estimated mean substitution rate of the M genes dataset was 1.3 × 10^−3^ (95% HPD=4.7 × 10^−4^-2.3 × 10^−3^) substitution per site per year.

### Recombination Analysis

**Figure 7:**
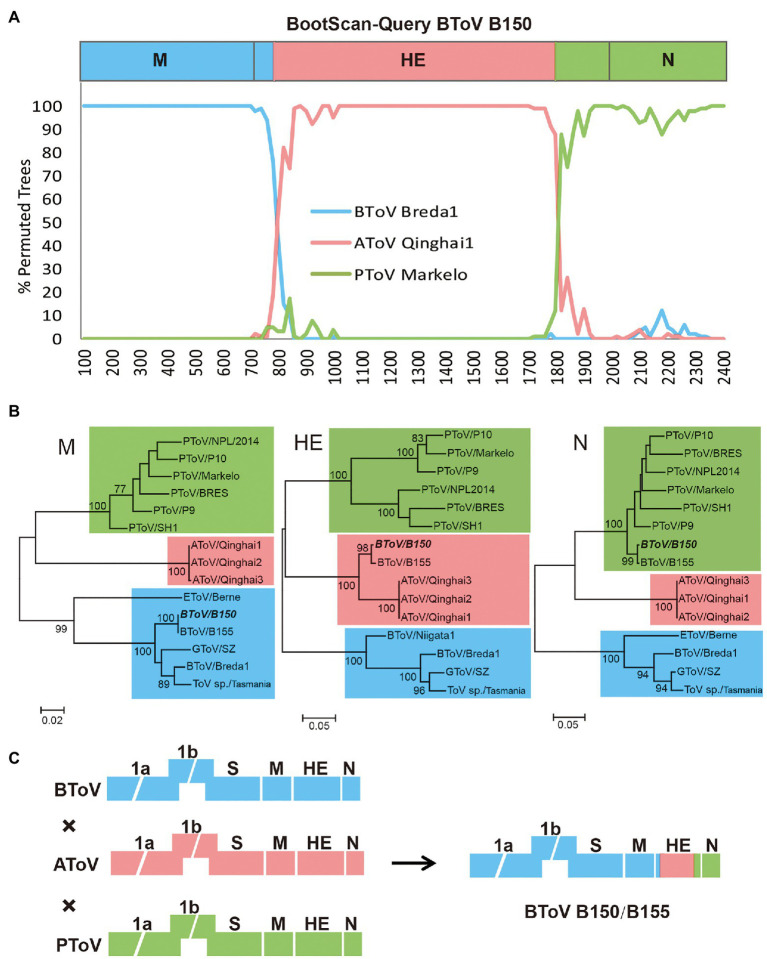
Recombination analysis among BToV, AToV, and PToV. **(A)** Bootscanning analysis was conducted with BToV B150 as the query, BToV Breda1, AToV Qinghai1, and PToV Markelo as the parental sequences; **(B)** Phylogenetic trees were constructed based on complete nucleotide sequences of M, HE, and N genes, respectively, by the maximum likelihood method. BToV B150 is italic bold. ToV strains cluster with BToV Breda1, AToV Qinghai1, and PToV Markelo are in the blue, pink, and green backgrounds, respectively. Bootstrap values (>70%) are shown at the branches. Scale bars below indicate the nucleotide substitutions per site; **(C)** Presumptive recombination event of BToV B150. Torovirus genomes are depicted schematically with different colors (BToV, AToV, and PToV were represented by blue, pink, and green, respectively).

To examine whether AToV has occurred in genetic recombination events of BToV B150 and B155, we performed a bootscanning analysis using the SimPlot software. As shown in Figure 7A, for the M gene, BToV B150 was more similar to BToV Breda1; for the HE gene, BToV B150 was more similar to BToV Breda1 from an approximate position the ATG codon to nt 80, more similar to AToV Qinghai1 from nt 81 to 1,100, and more similar to PToV Markelo from nt 1,101 to the end of the HE gene; for the N gene, BToV B150 was more similar to PToV Markelo (Figure 7A). The results of phylogenetic analyses based on M, HE, and N genes (Figure 7B) are consistent with the result of bootscanning analysis. These results are almost identical to that of the previous study (Smits et al., 2003) and demonstrated AToV is the unknown parental ToV of BToV B150 and B155 (Figure 7C).

### Morphological Observation

**Figure 8:**
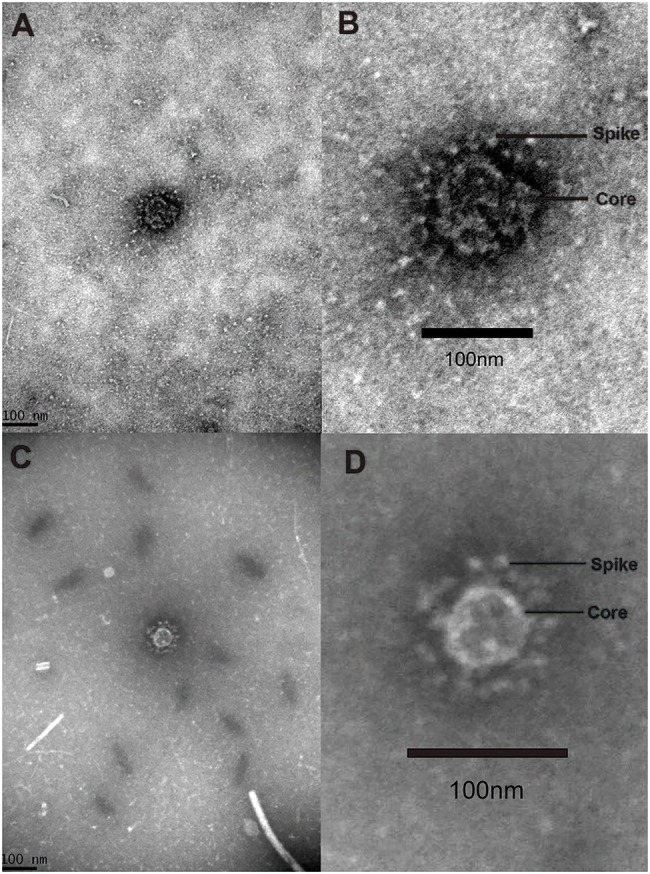
Virion. Negative stained ToV-like particles. **(A)** The low magnification TEM image of particle A; **(B)** the high magnification TEM image of particle A; **(C)** the low magnification TEM image of particle B; **(D)** the high magnification TEM image of particle B.

Three AToV-positive fecal samples were examined by transmission electron microscopy. Two spherical enveloped ToV-like particles with surface spikes of about 100nm in diameter were observed from one fecal sample (ID 61), which was one of the 160 fecal samples used for RNA sequencing. Each particle in a separate field of vision and was shown in Figure 8.

To justify considering these as toroviruses, therefore, we checked RNA sequencing data and found no contigs related to other viruses that have spikes in their particle surface including coronavirus in the family Coronavirdae, respirovirus, morbillivirus, henipavirus, avulavirus, rubulavirus, and pneumovirus in the family Paramyxoviridae and influenza virus A in the family Orthomyxoviridae (He et al., 2014). Only contigs related to toroviruses in the family *Tobaniviridae* were identified.

## Discussion

To date, there are three certain species of toroviruses, equine torovirus (EToV), bovine torovirus (BToV), and porcine torovirus (PToV) have been recognized in domestic ungulates. In this study, we have discovered three strains (Qinghai1, Qinghai2, and Qinghai3) of a novel ToV in a wild even-toed ungulate Tibetan antelope in Qinghai-Tibet plateau of China. We tentatively named the novel torovirus antelope torovirus (AToV) for its host as a species of antelope. Two ToV-like particles were observed directly from one AToV-positive fecal sample in separate visions by transmission electron microscopy.

AToV shares a similar genome organization with other species of ToVs, which contains six open reading frames and two non-coding regions at 5'-terminal and 3'-terminal. Eleven predicted conserved domains, including a newly found domain NiRAN (Snijder et al., 1990), were identified in pp1ab of AToV. Like other ToVs, the M^pro^ of AToV is a serine protease that resembled the M^pro^ of arterivirus; a putative slippery sequence was found in the overlapping region of ORF1a and ORF 1b; a predicted RNA pseudoknot with two loops and two stems locates downstream of the putative slippery sequence (Draker et al., 2006).

In phylogenetic trees conducted by amino acid sequences of ORF1a and ORF 1b of members of subfamilies Torovirinae, Piscanivirinae, and Orthocoronavirinae, three strains of AToV were clustered with members of the subfamily Torovirinae and formed a unique clade. In phylogenetic trees conducted with species of toroviruses within the subgenus *Renitovirus* using nucleotide sequences of complete genome, S (data not shown), M, HE, and N genes (Figures 5, 7B), three strains of AToV separated from other ToVs and formed an independent clade. Comparisons of the nucleotide and amino acid sequences of six ORFs between AToV Qinghai1 and other non-recombinant ToV strains, including strains of unassigned toroviruses GToV and ToV sp., showed that AToV Qinghai1 shared relatively low identity with other non-recombinant ToV strains, especially at the nucleotide level for S, M, HE, and N genes (in a range of 67.0–82.3%). Taken together, these data provide strong evidences that AToV represents a novel and distinct species in the subgenus *Renitovirus*. Evolutionary analysis based on the M genes showed tMRCA of ToVs occurred about 366years ago. The estimated mean substitution rate of the M genes dataset (1.3 × 10^−3^) is slightly lower than a previously calculated rate of substitutions (1.4 × 10^−3^) in BToV (Smits et al., 2003). We chose the M gene of ToV to conduct evolutionary analysis for (1) the length (702nt) of M gene is identical in all ToVs, (2) the data of full-length M genes of ToVs are adequate in Genbank, (3) only a few of full-length sequence data of complete genome, ORF1a, ORF1b, S gene, and N gene of ToVs are recorded in GenBank; using these sequence data may induce bias to evolutionary analysis, and (4) genetic recombination often occurred in the HE genes of ToVs (Smits et al., 2003; Cong et al., 2013), which may disturb the molecular clock for evolutionary analysis.

**Figure 9:**
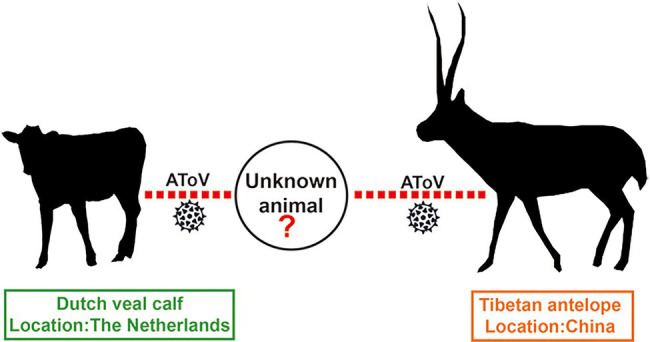
Possible transmission model of AToV. Red dotted lines indicated unknown routes of transmission for AToV among Tibetan antelope, unknown animal, and Dutch veal calf.

A previous study showed HE genes of BToV B150 and B155 were recombined by a BToV parent, a PToV parent, and an unknown ToV parent (Smits et al., 2003). Most strikingly, comparative, phylogenetic, and bootscanning analyses together indicated that AToV is the unknown ToV parent that involved in the recombinant events of chimeric HE genes of BToV B150 and B155. Segments of the HE gene of AToV were both found in fecal samples of Dutch veal calf (the host of BToV B150 and B155) in the Netherlands, and fecal samples of Tibetan antelope in China, which indicated the existence of AToV in these two animals and these two countries. Tibetan antelope, a wild and unique even-toed ungulate only in China, inhabits the harsh steppe areas at an elevation of 4,000–6,000km in the Qinghai-Tibet Plateau, is under China’s highest level of protection for poaching and used to be an endangered species that listed on the Red List of Threatened Species by the International Union for the Conservation of Nature and Natural Resources. There is no possible direct contact between Tibetan antelope in China and Dutch veal calf in the Netherlands. We speculated that there may exist an unknown animal that functioned as an intermediate host that connected these two animals for AToV spreading (Figure 9).

Our findings initially revealed Tibetan antelope is a natural reservoir host of toroviruses and indicated the diversity of toroviruses, which is far more than we know currently. Wild even-toed ungulates have been assessed as potential hosts of undiscovered viruses that may cause emerging zoonoses in the future (Olival et al., 2017). The proactive discovery of AToV of our study should be valued highly for (1) AToV was found in wild even-toed ungulate (Tibetan antelope) host and (2) cross-species transmission of AToV may occur among wild even-toed ungulate (Tibetan antelope), domestic even-toed ungulate (Dutch veal calf), and unknown intermediate host. The pathogenicity, animal infections, and geographic distribution of AToV should be evaluated in future studies.

## Data Availability Statement

The data presented in the study are deposited in the National Center for Biotechnology Information (NCBI), accession numbers MZ438674, MZ438675, MZ438676, and PRJNA751584.

## Ethics Statement

The animal study was reviewed and approved by the Ethics Committee of the National Institute for Communicable Disease Control and Prevention of Chinese Center for Disease Control and Prevention, and was performed according to Chinese Ethics Laws and Regulations.

## Author Contributions

JX conceived the research direction of this manuscript. XD performed the experiments, analyzed the experimental results, and drafted the manuscript. SL and GS supervised the sample collection. JX, JY, LL, and WZ reviewed the manuscript. All authors contributed to the article and approved the submitted version.

## Conflict of Interest

The authors declare that the research was conducted in the absence of any commercial or financial relationships that could be construed as a potential conflict of interest.

## Publisher’s Note

All claims expressed in this article are solely those of the authors and do not necessarily represent those of their affiliated organizations, or those of the publisher, the editors and the reviewers. Any product that may be evaluated in this article, or claim that may be made by its manufacturer, is not guaranteed or endorsed by the publisher.

## Footnotes

^1^ https://talk.ictvonline.org/taxonomy/
