# Supplementary material for: Characterization and Identification of a Novel Torovirus Associated With Recombinant Bovine Torovirus From Tibetan Antelope in Qinghai-Tibet Plateau of China
Source: Front Microbiol. 2021 Sep 6;12:737753. doi: 10.3389/fmicb.2021.737753 (PMC8451951; doi:10.3389/fmicb.2021.737753)
Supplement: Supplementary file 1 [file Data_Sheet_1.PDF]

Supplemental Table S1. A set of RT-PCR primers used for the amplification of the AToV genome

Primer name	Primer sequence (5' – 3')	Location or Illustration
OligodT-Anchor Primer	Provided by 5'/3' RACE Kit	For synthesis of 5' and 3' ends
PCR Anchor Primer	Provided by 5'/3' RACE Kit	For synthesis of 5' and 3' ends
SP1	CTAAACACTGGTCCAAAAGC	938-957
SP2	GGTGTCCGAAATGGGCAAAT	826-845
F1	TTAGTTGATTTTGAAGCCTTG	1-21
R1	CTAAACACTGGTCCAAAAGC	938-957
NR1	GGTGTCCGAAATGGGCAAAT	826-845
F2	AAACACCGTAGACGCCATTC	726-745
R2	GTTCAGTAGGTTCGGCAATA	3914-3933
NR2	TTCTCACAGTATGACAGCCC	3792-3811
F3	TGTGCTTGTAGCGGTGAGTG	3696-3715
R3	CCTCGGTCTGGGTGTTTCAA	6790-6809
NR3	GACCACGCTCTCTCAAAACC	6752-6771
F4	GAGTGGGCTATGATTGCTTA	6550-6569
NF4	TTTGGTCAGGACAAGAGTGG	6636-6655
R4	GAGCCAAGACAAACACAACAC	9844-9864
F5	CCCTTTGAGGTTGAGCATTC	9576-9595
R5	AGGGTAGGCAGACAATGGTT	12904-12923
NR5	TGAGGTAAGAAGGCAAGATG	12724-12743
F6	ATGTCATCTTTGAAGGTTAGGA	12684-12705
R6	CACAAACCATAGTATCCAACAT	15926-15947
NR6	GAAAGGGGAAATGTCCTATCAC	15816-15837
F7	TCATCTTGCTCCTTTATTGC	15614-15633
NF7	GATAGGACATTTCCCCTTTCTT	15818-15839
R7	TAACTACCACTACGCATCAAAC	18798-18819
F8	AATCAGCATTGCGTGGTTTA	18552-18571
R8	TAGCGAGATTGTTGATAGAGGG	21932-21958
NR8	GGTGACTGAAAGGACTTAGATA	21878-21899
F9	TGCTACCTACCAAACAGTCACCT	21500-21522
R9	GGCAAATCATACTGTAGACTGGT	24958-24980
NR9	TGGCATCTGTAACAAGAGGCAAT	24822-24844
F10	TGGAGCAAATAACACAACAG	24536-24555
NF10	AAGTGGGAACTGTCAACCTA	24768-24787
R10	TCTCATTTGCCATCATAAGC	28042-28061
F11	GGCAGTTCAGAGTATTTGGTC	27303-27323
NF11	GCCGTCAGTCTAACAATCAGT	27993-28013
R11	AGCTGCTTTTTACTACTTCTGG	28417-28438
SP5	TTTGAGCCGCCAGAGTAGAT	27916-27935

Supplemental Table S2. $\log_{10}$ Bayes factors of different clock model with Constant Size tree prior for BEAST analysis

Model	Strict clock	Lognormal relaxed clock	Exponential relaxed clock	Random local clock
Strict clock	-	-.5.42	-12.07	-1.93
Lognormal relaxed clock	5.42	-	-6.65	3.50
Exponential relaxed clock	**12.07**	**6.65**	-	**10.15**
Random local clock	1.93	-3.50	-10.15	-

Supplemental Table S3. $\log_{10}$ Bayes factors of Exponential relaxed clock with different tree prior for BEAST analysis

Tree prior	Constant Size	Exponential Growth	Logistic Growth	Bayesian Skyline	Extended Bayesian Skyline plot	GMRF Bayesian Skyride
Constant Size	-	**0.07**	**0.57**	**0.37**	**0.06**	**0.62**
Exponential Growth	-0.07	-	0.47	0.30	-0.01	0.55
Logistic Growth	-0.57	-0.47	-	-0.20	-0.51	0.06
Bayesian Skyline	-0.37	-0.30	0.20	-	-0.311	0.26
Extended Bayesian Skyline plot	-0.06	0.01	0.51	0.311	-	0.57
GMRF Bayesian Skyride	-0.62	-0.55	-0.06	-0.26		-

Supplemental Table S4. Strains of toroviruses used in this study

Virus species	Description of sequence	Strain	Accession number	Year of collection	Locality
BToV	Genome	Breda1	AY427798	1979	Canada
		Ishikawa/2010	LC088094	2010	Japan
		Kagoshima/2014	LC088095	2014	Japan
		SC-1	MN073058	2018	China
		SC-2	MN073059	2018	China
		yak-XZ01	MN882587	2018	China
	Partial genmoe	HT1-TUR	MG957145	2016	China
		HT2-TUR	MG957146	2016	China
	S gene	B145	AJ575373	1998	Netherland
		Miyagi-2006TI/E	AB526862	2006	Japan
		Gifu-2007TI/E	AB526863	2007	Japan
		Hokkaido-2008TI/E	AB526864	2008	Japan
		Gifu-2009TI/E	AB526865	2009	Japan
		Aichi/2004	AB526866	2004	Japan
	M gene	B155	AJ575377	1998	Netherland
		B150	AJ575376	1998	Netherland
		B145	AJ575375	1998	Netherland
		BToV	AJ575374	1990	Netherland
	HE gene	Breda2	Y10866		Netherland
		B6	AJ575378	1990	Italy
		B145	AJ575379	1998	Netherland
		B150	AJ575380	1998	Netherland
		B155	AJ575381	1998	Netherland
		Niigata1	AB661456	2007	Japan
		Niigata2	AB661457	2007	Japan
		Niigata3	AB661458	2008	Japan
		Niigata3(TC)	AB661459	2008	Japan
		Aichi/2004	AB661460	2004	Japan
		Aichi/2004(LIC)	AB661461	2004	Japan
EToV	Genome	Berne	MG996765	1972	Switzerland
	ORF1a	Berne	DQ310701	1972	Switzerland
	ORF1b	Berne	DQ310701	1972	Switzerland
	S gene	Berne	X52506	1972	Switzerland
	M gene	Berne	X52505	1972	Switzerland
	N gene	Berne	D00563	1972	Switzerland
GToV	Genome	SZ	NC_034976	2012	China
ToV sp.	Genome	harrisii/2016/DN128115	MK521914	2017	Australia
PToV	Genome	NPL/2014	KM403390	2014	Japan
		SH1	JQ860350	2010	Japan

	ZJU39	MT684462	2016	China
	HB1	MH603532		China
	Ibaraki	LC483442	2018	Japan
	GER	LT900503		Germany
	L00926-K20			
S gene	07-56-22	GU196786	2007	Korea
	Markelo	AJ575372	1995	Netherland
M gene	SC-2013-I2	HG764770	2013	China
	SC-2013-I1	HG764769	2013	China
	SC-2012-K	HG764768	2012	China
	SC-2012-J	HG764767	2012	China
	SC-2012-L2	HG764766	2012	China
	SC-2012-L1	HG764765	2012	China
	SC-2012-H2	HG764764	2012	China
	SC-2012-H1	HG764763	2012	China
	SC-2012-G	HG764762	2012	China
	SC-2011-F	HG764761	2011	China
	SC-2011-E	HG764760	2011	China
	SC-2012-D2	HG764759	2012	China
	SC-2012-D1	HG764758	2012	China
	SC-2012-C2	HG764757	2012	China
	SC-2012-C1	HG764756	2012	China
	SC-2011-B2	HG764755	2011	China
	SC-2011-B1	HG764754	2011	China
	SC-2011-A2	HG764753	2011	China
	SC-2011-A1	HG764752	2011	China
	07-109-11	GU181244	2007	Korea
	07-109-14	GU181241	2007	Korea
	07-109-15	GU181240	2007	Korea
	07-55-4	GU181250	2007	Korea
	07-55-5	GU181249	2007	Korea
	07-56-11	GU181248	2007	Korea
	07-56-14	GU181247	2007	Korea
	07-56-23	GU181245	2007	Korea
	07-109-12	GU181243	2007	Korea
	07-109-13	GU181242	2007	Korea
	P10	AJ575371	1999	Italy
	P9	AJ575370	1996	Italy
	P4	AJ575369	1990	Italy
	Markelo	AJ575368	1995	Netherland
	BRES	FJ232069	2002	Italy
HE gene	52_11	GU299777	2005	Spain
	52_7	GU299776	2005	Spain
	14_7	GU299775	2005	Spain

	13_11	GU299774	2005	Spain
	12_11	GU299773	2005	Spain
	07-109-11	GU181255	2007	Korea
	07-109-12	GU181254	2007	Korea
	07-109-13	GU181253	2007	Korea
	07-109-14	GU181252	2007	Korea
	07-109-15	GU181251	2007	Korea
	07-55-4	GU187335	2007	Korea
	07-55-5	GU187334	2007	Korea
	07-56-11	GU187333.	2007	Korea
	07-56-14	GU187332	2007	Korea
	07-56-22	GU187331	2007	Korea
	07-56-23	GU187330	2007	Korea
	P78	AJ575367	2000	Hugary
	P9	AJ575365	1996	Italy
	Markelo	AJ575363	1995	Netherland
	BRES	FJ232070	2002	Italy
	P10	AJ575366	1999	Italy
N gene	BRES	FJ232068	2002	Italy
	P78	AJ575362	2000	Hugary
	P10	AJ575361	1999	Italy
	P9	AJ575360	1996	Italy
	P4	AJ575359	1990	Italy
	Markelo	AJ575358	1995	Netherland
